# Human Sperm Quality and Metal Toxicants: Protective Effects of some Flavonoids on Male Reproductive Function

**DOI:** 10.22074/ijfs.2016.4912

**Published:** 2016-06-01

**Authors:** Mostafa Jamalan, Mohammad Ali Ghaffari, Pooneh Hoseinzadeh, Mahmoud Hashemitabar, Majid Zeinali

**Affiliations:** 1 Abadan School of Medical Sciences, Abadan, Iran; 2 Cellular and Molecular Research Center, Ahvaz Jundishapur University of Medical Sciences, Ahvaz, Iran; 3 Department of Biochemistry, Ahvaz Jundishapur University of Medical Sciences, Ahvaz, Iran; 4 Department of Anatomical Sciences, Ahvaz Jundishapur University of Medical Sciences, Ahvaz, Iran; 5 Biotechnology Research Center, Research Institute of Petroleum Industry (RIPI), Tehran, Iran

**Keywords:** Metal Toxicity, Sperm Motility, Lipid Peroxidation, Flavonoids, Semen Quality

## Abstract

**Background:**

Metals can cause male infertility through affection of spermatogenesis and sperm quality. Strong evidences confirm that male infertility in metal-exposed humans is mediated via various mechanisms such as production of reactive oxygen species (ROS). Flavonoids have antioxidant and metal chelating properties which make them suitable candidates for neutralizing adverse effects of metals on semen quality. In the current study, we have evaluated the effects of five types of flavonoids (rutin, naringin, kaempferol, quercetin, and catechin) on recovery of sperm motility and prevention of membrane oxidative damage from aluminum chloride (AlCl_3_), cadmium chloride (CdCl_2_), and lead chloride (PbCl_4_).

**Materials and Methods:**

In this experimental study, motility and lipid peroxidation of metalexposed sperm was investigated in the presence of different concentrations of five kinds of flavonoids. Malondialdehyde (MDA) production was assessed as a lipid peroxidation marker.

**Results:**

Aluminum chloride (AlCl_3_), cadmium chloride (CdCl_2_), and lead chloride (PbCl_4_) diminished sperm motility. Treatment of metal-exposed sperm with rutin, naringin, and kaempferol attenuated the negative effects of the metals on sperm motility. Quercetin and catechin decreased the motility of metal-exposed sperm.

**Conclusion:**

Based on the MDA production results, only AlCl_3_ significantly induced lipid peroxidation. Treatment with rutin, naringin, and kaempferol significantly decreased MDA production.

## Introduction

Metals are one of the main constituents of an industrialized lifestyle that have a wide range of applications. Metals such as lead (Pb), aluminum (Al) and cadmium (Cd) induce toxicity in humans and other living organisms by impacting enzyme activity and generation of free radical production. However, in terms of their unique characteristics, their applications are expansive, even in medical and drug industries (1,2). 

**Fig.1:**
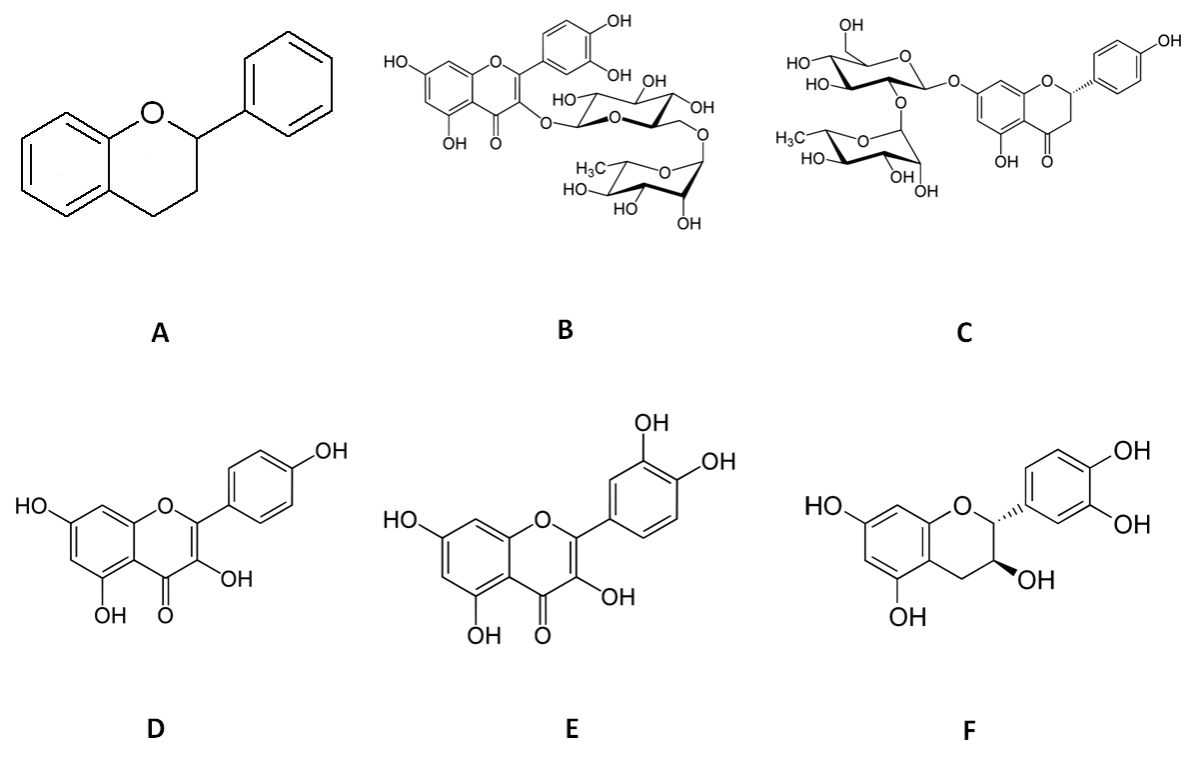
Chemical structure of flavonoids. A. Basic structure of a flavonoid with two benzene rings and a heterocyclic pyran ring as the linker. Chemical structures of: B. Rutin, C. Naringin, D. Kaempferol, E. Quercetin, and F. Catechin

Metals can affect male and female fertility by induction of reactive oxygen species (ROS) production. Therefore, antioxidant therapy that inhibits metal-induced toxicity is under active investigation (3). Flavonoids are a broad group of natural antioxidant compounds with flavan nucleus and a benzo-ƴ-pyrone structure. These compounds are low molecular weight polyphenols ubiquitously synthesized by green plants that may show various pharmacological attributes according to their chemical structures (4). Direct antioxidant effects and the ability of flavonoids to chelate metal ions have been previously researched (5,7). Researchers report the existence of a cardioprotective role (8,9) and free radical scavenging potential of flavonoids (10). Until now, over 4000 natural flavonoids have been identified in leaves, seeds, barks, and flowers of different plants (11). Protection against ultraviolet (UV) light, pathogens, herbivores, and the attraction of pollinating insects are major proposed roles for flavonoids in various plants (12,14). Flavonoids can occur both in the free form and as glycosides. Their structure is composed of a basic C_6_-C_3_-C_6_phenyl-benzopyran backbone (Fig .1). The position of the phenyl ring relative to the benzopyran moiety, oxidation of central ring, hydroxylation profile, and degree of polymerization determine chemical properties of a flavonoid (15). 

ROS induce cellular membrane instability (16), destruction of DNA structures, and promotion of transformation, (17) ultimately resulting in cellular aging (18), mutagenesis (17), carcinogenesis (19), induction of coronary heart disease (CHD) (4), and infertility (20). In addition to ROS, nitrogen reactive species (NOS) can cause cardiovascular diseases (CVD) through oxidation of LDL particles (21,22) and increased release of matrix metalloproteinase-2 (MMP-2) in the coronary effluent (23). Based on the scientific findings, a flavonoid-rich diet is highly recommended to decrease CVD and other ROS-/ NOS-induced myocardial injuries (4). 

Recent interest in flavonoids arises from the potential health benefits attributed to the antioxidant activities of these polyphenolic compounds. Functional hydroxyl groups in flavonoids mediate their antioxidant effects by scavenging free radicals and/ or by chelating metal ions (4,11). The chelating of metals can be crucial in prevention of radical generation which damage target biomolecules (11). In the current study, we have evaluated the effects of five types of flavonoids (rutin, naringin, kaempferol, quercetin, and catechin) on recovery of sperm motility and prevention of membrane oxidative damage from aluminum chloride (AlCl_3_), cadmium chloride (CdCl_2_ ), and lead chloride (PbCl_4_). 

## Materials and Methods

### Materials

For this experimental study, AlCl_3_ , CdCl_2_ , PbCl_4_ , naringin, kaempferol, and quercetin were obtained from Merck (Darmstadt, Germany). Rutin, catechin and the remainder of chemicals and reagents used in this research were purchased from SigmaAldrich (St. Louis, MO, USA). 

### Sample collection and preparation of sperm suspension

Sperm samples considered compatible to the world health organization (WHO) reference value for human semen (volume ≥3.0, sperm concentration/ ml ≥50×10^6^, forward motility ≥60%, and atypical forms ≤40%) (24) were collected and pooled from 40 healthy, non-smoking volunteers, that resided in Ahvaz, Khuzestan Province, Iran. We compared the effects of flavonoides on motility and lipid peroxidation of metal-exposed sperms using laboratory studies. The Institutional Ethics Committee of Ahvaz University of Medical Sciences reviewed and approved the protocol. All participants in the current study signed informed consents. Collected sperm samples were separated from semen plasma for assessment of clinical attributes by washing three times with an equal volume of M ^6^solution and subsequent centrifugation for 10 minutes at 1600 g (25). M_6_ solution contained (per liter, pH=7.4): 0.55% NaCl, 0.03% KCl, 0.019% CaCl_2_ , 0.016% K_3_PO_4_, 0.029% MgSO_4_, 0.031% NaHCO_3_, 0.496% HEPES, 0.26% sodium lactate, 36×10^-4^% sodium pyruvate, 0.11% glucose, 0.4% bovine serum albumin, 60×10^-4^% penicillin, and 50×10^-4^% streptomycin. Separated pellets were suspended in M ^6^solution at a density of 100 million sperm/ml and freshly were used. Sperm counts were performed by a MMC-SK Sperm Counting Chamber (Saint Petersburg, Russia). 

### Incubation of sperm samples with aluminum chloride, cadmium chloride, and lead chloride

We evaluated the effects of AlCl_3_ , CdCl_2_ , and PbCl_4_ on sperm motility and lipid peroxidation of sperm cells at different concentrations (125 µM, 250 µM, 500 µM, 1 mM, and 5 mM) of the metal salts. The metal salt solutions were prepared in M ^6^solution. Sperm samples were incubated in the presence of defined concentrations of these metals for 2 hours at 37˚C. From the examined concentrations of metals, we selected those that significantly impacted sperm motility for additional experiments with the flavonoids (P≤0.05). 

### Effects of flavonoids on the motility of metalexposed sperm

Sperm samples were treated for 2 hours at 37˚C with AlCl_3_ (1.0 mM), CdCl_2_ (500 µM) or PbCl_4_ (250 µM) in the presence of various concentrations (25, 50, 100, 200, 500, and 1000 µM) of rutin, naringin, kaempferol, quercetin, and catechin. Subsequently, we assessed sperm mobility by MMC Sperm. In order to increase solubility, all flavonoids were solvated in a 1:1 (v/v) of Dimethyl sulfoxide (DMSO): M_6_solution prior to their treatment of the sperm cells. 

### Effects of flavonoids on lipid peroxidation of metal-exposed sperm

Induction of lipid peroxidation was evaluated in sperm samples in the presence of various concentrations of AlCl_3_ , CdCl_2_ , and PbCl_4_ . Between treated groups, sperm samples treated with 20 mM of AlCl_3_ were simultaneously incubated with 25 µM, 50 µM, 100 µM, 200 µM, 500 µM, and 1 mM each of rutin, naringin, kaempferol, quercetin, and catechin for 2 hours at 37˚C. After incubation, we assessed for lipid peroxidation of the sperm cells according to the indicated approach. 

### Analytical methods

#### Assessment of sperm motility

Evaluation of sperm motility was performed by MMC Sperm (MultiMedia Catalog Sperm). MMC Sperm is an automated image analysis software package for sperm quality analysis according to parameters recommended by the WHO laboratory manual (26). 

#### Measurement of lipid peroxidation 

Lipid peroxidation was measured using malondialdehyde (MDA) and thiobarbituric acid-reactivity (27,28). Briefly, 50 µl of 0.2% butylated hydroxytoluene (dissolved in ethanol) and 1.0 ml of 15% aqueous trichloroacetic acid were successively added to 2.0×10^7^sperm. The mixture was then centrifuged at 4000 g for 15 minutes at 4˚C. An aliquot of 500 μl of the deproteinized supernatant was added to 1.0 ml thiobarbituric acid (0.375% in 0.25 M HCl) and the mixture was heated at 100˚C for 20 minutes. After cooling, the solution was analyzed by a spectrophotometer at 532 nm. 

### Statistical analysis

All treatments were performed in triplicate. Each experiment was run at least three times. Results were expressed as mean ± SE. Significance of difference between treatment groups was determined by the student’s t test. P<0.05 was considered statistically significant. 

## Results

### Effects of aluminum chloride, cadmium chloride, and lead chloride on sperm motility

**Fig.2:**
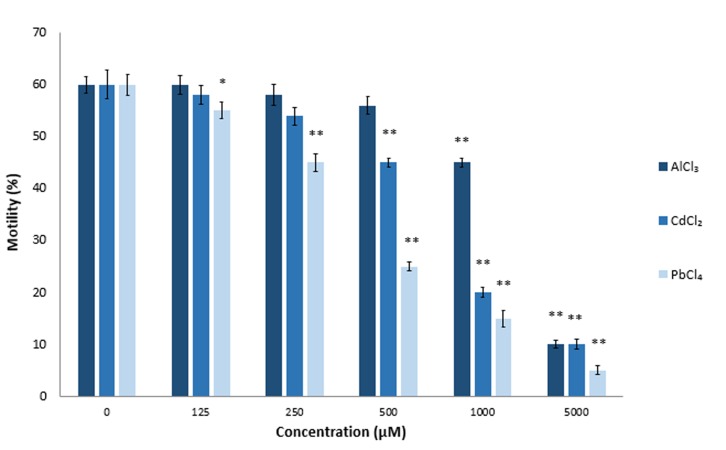
Effects of aluminum chloride (AlCl_3_), cadmium chloride (CdCl_2_), and lead chloride (PbCl_4_) on sperm motility. We evalu- ated the effects of these compounds on sperm motility at differ- ent concentrations (125 μM, 250 μM, 500 μM, 1 mM, and 5 mM) of metal salts. Sperm samples were incubated in the presence of the defined concentrations of metals for 2 hours at 37˚C. *; P<0.05 and **; P<0.01 compared to the untreated control.

AlCl_3_ is an abundant metal in the earth which has toxic effects. High concentrations of AlCl_3_ induce free radical-mediated cytotoxicity and can be toxic for the male reproductive system (29,30). In previous studies, it has been shown that treatment with AlCl_3_ could decrease ejaculate volume, sperm concentration, and sperm motility (31). CdCl_2_ is a wellknown nephrotoxin and carcinogen (32,33) that can induce ROS production. Exposure to CdCl_2_ may result in decreased sperm concentration, diminished sperm motility, creation of abnormal forms of sperm following long-term exposure to CdCl_2_ (3,34), and infertility in treated male mice (35). PbCl_4_ poisoning can result in decreased sperm motility. A number of reports discuss DNA fragmentation in sperm cells exposed to this metal *in vitro* (36). Our *in vitro* studies have confirmed the above mentioned findings where different concentrations of AlCl_3_ , CdCl_2_ and PbCl_4_ significantly decreased sperm motility (P≤0.05, Fig .2). Mean sperm motility after a 2-hour incubation period in the presence of 5.0 mM AlCl_3_ , CdCl_2_, and PbCl_4_ were 93% (AlCl_3_), 75% (CdCl_2_ ), and 41% (PbCl_4_) less than the control groups. As seen in Figure 2, the effect of Pb on sperm motility was higher at the same concentrations of the three tested metals AlCl_3_ , at the 1.0 mM concentration, significantly affected sperm motility (P≤0.0013). The 500 µM concentration of CdCl_2_ significantly affected sperm motility (P≤0.032), whereas PbCl_4_ significantly affected motility at the 250 µM (P≤0.0005) concentration (Fig .2). The adverse effects of all three metals on sperm motility were completely dose-dependent. 

### Effects of flavonoids on motility of aluminum chloride-exposed sperm

**Fig.3:**
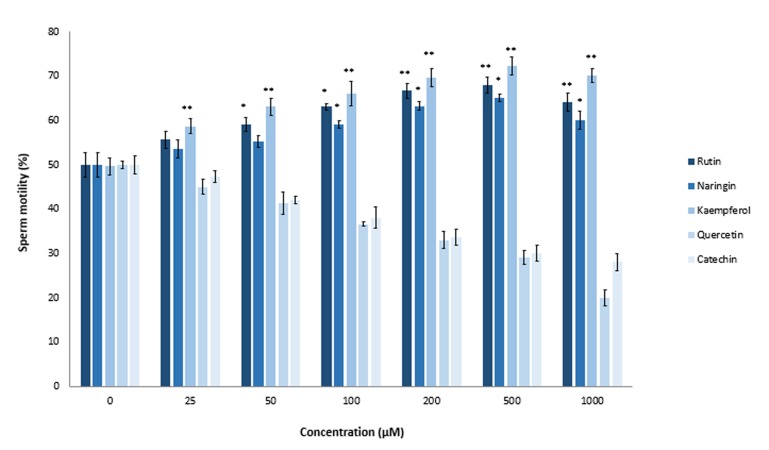
Effects of rutin, naringin, kaempferol, quercetin, and catechin on aluminum chloride (AlCl_3_)-exposed sperm. Sperm samples were treated for 2 hours at 37˚C with AlCl_3_ (1.0 mM) in the presence of various concentrations (25, 50, 100, 200, 500, and 1000 μM) of rutin, naringin, kaempferol, quercetin, and catechin. Sperm mobility was assessed by MMC Sperm. *; P<0.05 and **; P<0.01 compared to the flavonoid untreated control.

Previous studies reported an *in vitro* protective effect of ascorbic acid (vitamin C) and tocopherol (vitamin E) on AlCl_3_ -treated sperm (31,37). As seen in Figure 2, 1000 µM of AlCl_3_ significantly decreased sperm motility by 15% (P≤0.0013). Therefore, we used this concentration for additional studies with flavonoids. We used different concentrations of rutin, naringin, kaempferol, quercetin, and catechin for motility recovery of AlCl_3_ -exposed sperm. Compared to the untreated control group, rutin increased sperm motility by 9% at the 50 µM concentration and 18% at the 200 µM concentration. Naringin, at a final concentration of 100 µM, significantly increased sperm motility by 9% (P≤0.038). There was a gradual increase in recovery of sperm motility when the concentration of naringin increased to 500 µM (Fig .3). Kaempferol showed the most protective effect of all the tested flavonoids. There was 10% recovery of sperm motility at the kaempferol concentration of 25 µM. On the other hand, effects of quercetin and catechin on the sperm mobility completely differed from the other tested flavonoids rutin, naringin and kaempferol. The antioxidants, quercetin and catechin did not protect sperm cells from heavy metal-mediated damages; rather, they showed inhibitory effects on sperm motility. When we increased the concentrations of quercetin and catechin from 0 to 1000 µM, there was a gradual decrease in sperm motility compared to the untreated control group. Mean motility of AlCl_3_ -exposed sperm after a 2 hours incubation period in the presence of 1000 µM quercetin was 22% and for catechin, it was 28%. 

### Effects of flavonoids on motility of cadmium chloride-exposed sperm

**Fig.4:**
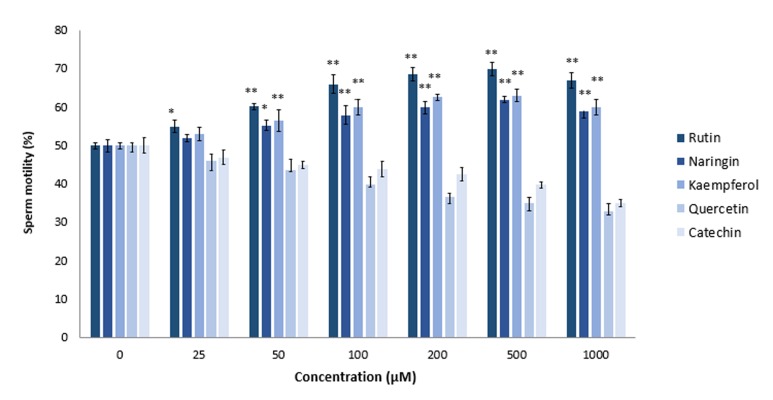
Effects of rutin, naringin, kaempferol, quercetin, and catechin on cadmium chloride (CdCl_2_ )-exposed sperm. Sperm samples were treated for 2 hours at 37˚C with CdCl_2_ (500 μM) in the presence of various concentrations (25, 50, 100, 200, 500, and 1000 μM) of rutin, naringin, kaempferol, quercetin, and catechin. Sperm mobility was assessed by MMC Sperm. *; P<0.05 and **; P<0.01 compared to the flavonoid untreated control.

Previous studies by El-Demerdash et al. (3) in male rats showed beneficial effects of vitamin E and β-carotene in reducing the toxic effects of CdCl_2_ on the male reproductive system. In the current study, we observed that treatment with rutin, naringin and kaempferol resulted in recovery of motility in CdCl_2_ exposed sperm cells. Our results showed that rutin, naringin, and kaempferol at 25-500 μM significantly increased (P≤0.05) motility of CdCl_2_ -exposed sperm cells in a dose-dependent manner (Fig .4). In contrast, quercetin and catechin did not induce any protective effect against CdCl_2_ toxicity; they reduced the motility of CdCl_2_ -exposed sperm compared to the untreated control samples (Fig .4). These results disagreed with an in vivo study by Farombi et al. (38) about the antioxidative nature of quercetin. They showed that administration of the biflavonoid, kolaviron, or quercetin prevented Cd-mediated decreased sperm motility in adult male rats. Other researchers reported the positive effects of quercetin on sperm capacity under both *in vitro* and in vivo conditions (39). Supplementation of quercetin restored the decrease in glutathione (GSH) level, and superoxide dismutase (SOD) and GSH peroxidase activities in Cd-exposed mice. This discrepancy between *in vitro* and in vivo results might be attributed to the difference in quercetin exposure time or to *in situ* metabolic alteration of quercetin (40). 

### Effects of flavonoids on motility of lead chlorideexposed sperm

**Fig.5:**
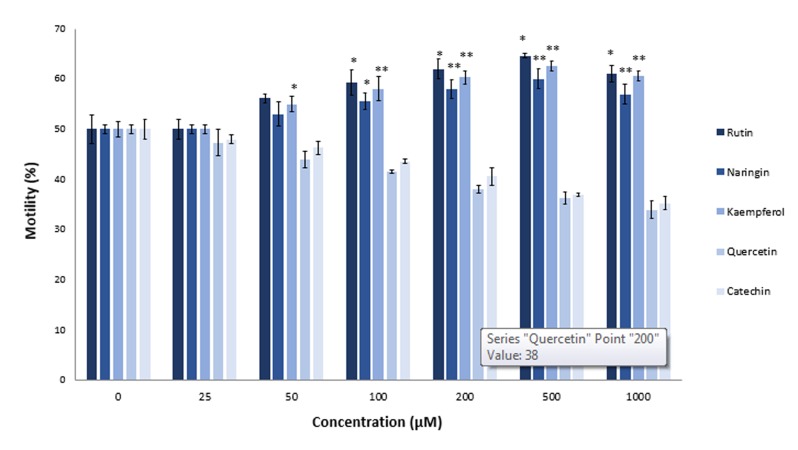
Effects of rutin, naringin, kaempferol, quercetin, and catechin on lead chloride (PbCl_4_)-exposed sperm. Sperm samples were treated for 2 hours at 37˚C with PbCl_4_ (250 μM) in the presence of various concentrations (25, 50, 100, 200, 500, and 1000 μM) of rutin, naringin, kaempferol, quercetin, and catechin. Sperm mobility was assessed by MMC Sperm. *; P<0.05 and **; P<0.01 compared to flavonoid untreated control.

Toxic effects of PbCl_4_ on sperm quality, motility, DNA fragmentation, and acrosome reaction have been investigated extensively in mice and humans (36,41,44). According to our results (Fig .2), PbCl_4_ compared to AlCl_3_ and CdCl_2_ had more adverse effects on sperm motility at the 0.125 to 5.0 mM concentrations. We used the 250 µM concentration of PbCl_4_ for additional experiments with flavonoids. Quercetin and catechin decreased motility of PbCl_4_ -exposed sperm cells in a dose-dependent manner. However, as seen in Figure 5, the 500 µM concentration of rutin, naringin, and kaempferol significantly increased sperm motility to 65% (rutin), 60% (naringin) and 63% (kaempferol). Rutin was more efficient in fortifying sperm cells against PbCl_4_-induced harmful attacks. 

### Sperm lipid peroxidation in the presence of aluminum chloride, cadmium chloride and lead

**Fig.6:**
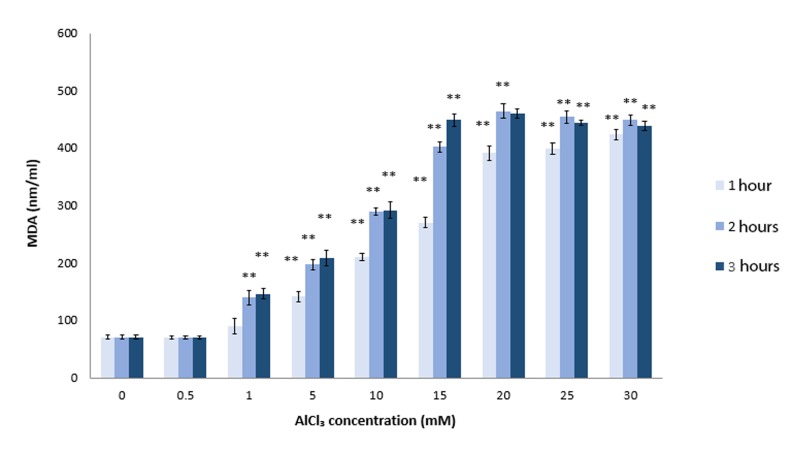
Sperm lipid peroxidation in the presence of aluminum chloride (AlCl_3_). Sperm samples were treated with AlCl_3_ (20 mM) for 2 hours at 37˚C. After incubation, we assessed the amount of lipid peroxidation of the sperm cells with MDA. **; P<0.01 compared to the untreated control group and MDA; Malondialdehyde.

chloride Sperm membranes are rich in polyunsaturated fatty acids (PUFAs) (45). Previous in vivo studies have demonstrated that Al could increase peroxidation of PUFAs in sperm samples (31,46). The presence of a high level of PUFA in the sperm plasma membrane is required for membrane fusion events associated with fertilization. Loss of fluidity as a result of lipid peroxidation can diminish the rates of sperm-oocyte fusion (47). Our *in vitro* studies have shown that AlCl_3_ at concentrations higher than 0.5 mM significantly induced MDA production after 1 hour of incubation (P≤0.0008, Fig .6). MDA is an end-product of enzymatic and oxygen radical-initiated oxidative decomposition of PUFAs and most frequently used as an indicator of lipid peroxidation. We have shown that the effect of AlCl_3_ on sperm lipid peroxidation was doseand time-dependent (Fig .6). There were no significant changes in sperm MDA formation observed following incubation with 0.5-30 mM of CdCl_2_ or PbCl_4_ (data not shown). Therefore, we only investigated the effects of flavonoids on MDA formation in AlCl_3_ -exposed sperm cells. 

### Effects of flavonoids on lipid peroxidation of aluminum chloride-exposed sperm

**Fig.7:**
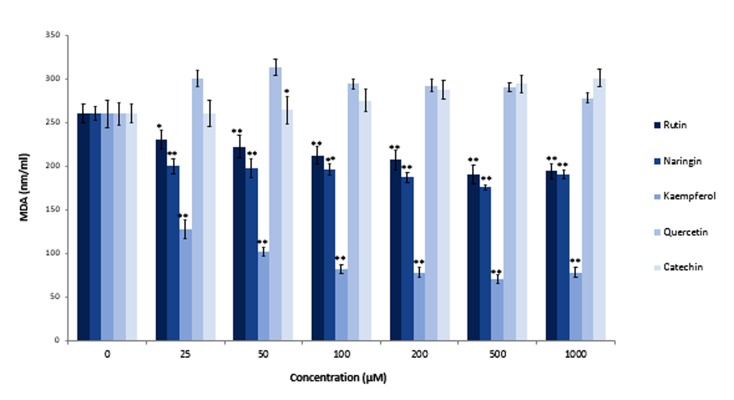
Effects of rutin, naringin, kaempferol, quercetin, and catechin on lipid peroxidation of aluminum chloride (AlCl_3_)-exposed sperm. Sperm samples were treated with AlCl_3_ (20 mM) and simultaneously incubated with different concentrations of rutin, naringin, kaempferol, quercetin, and catechin for 2 hours at 37˚C. After incubation, we assessed the lipid peroxidation of sperm cells with MDA. *; P<0.05, **; P<0.01 compared to the flavonoid untreated control group and MDA; Malondialdehyde.

Researchers previously reported the protective effect of ascorbic acid as an antioxidant against induction of lipid peroxidation by AlCl_3_ in sperm cells (46). However, to the best of our knowledge there was no report about the protective effect of flavonoids against lipid peroxidation in Alexposed sperm cells. Moretti et al. showed that quercetin, rutin and, to a lesser extent, naringenin, significantly decreased tert-butyl hydroperoxide induced lipid peroxidation in human sperm (48). Their studies indicated that epicatechin was not efficacious as an antioxidant to protect sperm cells against oxidants. Our investigations showed that kaempferol was the most effective amongst the tested products in protection of sperm cells against AlCl_3_ -induced lipid peroxidation (Fig .7). Kaempferol, at a concentration of 100 µM, reduced MDA production from 250 nmol/ml (in untreated cells) to approximately 80 nmol/ml. Naringin and rutin were less effective in protection of AlCl_3_ -exposed sperm cells against lipid peroxidation compared to kaempferol. We observed that quercetin and catechin did not protect sperm. Quercetin, as an antioxidant, did not protect sperm cells against lipid peroxidation; rather, it had inhibitory effects on sperm motility. Khanduja et al. (49) have reported a significant decrease in sperm Ca ^2+^-ATPase activity following quercetin treatment. Ca ^2+^-ATPase is the responsible enzyme that provides energy for progressive movement of sperm cells. Inhibition of Ca ^2+^-ATPase activity has been shown to result in Ca ^2+^accumulation in the cells and blockage of the sperm motility apparatus (50). 

## Discussion

The impact of heavy metal toxicity, even at low concentrations, on the male reproductive system has been extensively investigated and confirmed (51,54). Sperm motility depends on the synchronized actions of proteins, sugars, ions, and small organic molecules. It is one of the main factors that facilitates the journey of sperm toward the egg and the subsequent fertilization process (55). Defects in sperm motility are a common reason for infertility in humans (56). In the current study we have shown that AlCl_3_ , CdCl_2_ and PbCl_4_ significantly affected sperm motility. PbCl_4_ had the most toxic effect. 

Infertility due to metal toxicity usually occurs as a result of ROS induction (57). Therefore, antioxidant therapy is a promising strategy for treatment of individuals with heavy metal poisoning (58). Among natural antioxidants, flavonoids are more likely to exert protective activities against metal toxicity compared to carotenoids and vitamin E (37,59). Based on our results, three flavonoids, rutin, naringin, and kaempferol have been shown to restore motility of AlCl_3_ -, CdCl_2_ -, and PbCl_4_ exposed sperm cells. The other two flavonoids, catechin and quercetin, had no positive effects on motility of metal-exposed sperm; rather, they decreased sperm motility compared to untreated control samples. 

We conducted additional research on the protective effects of flavonoids as antioxidant agents against heavy metal-induced lipid peroxidation. MDA formation was assessed in AlCl_3_ -exposed sperm cells treated with the five above mentioned flavonoids. Among flavonoids, quercetin due to its free radical scavenging and metal chelating abilities has been extensively investigated (60). However, according to the obtained results, quercetin and catechin did not protect sperm cells from ROS-mediated damages. They adversely affected sperm motility. Inhibition of sperm motility without considerable effects on peroxidation of PUFAs would indicate involvement of other inhibitory mechanisms. In contrast, increased motility of Alexposed sperm cells treated with rutin, naringin and kaempferol was accompanied by decreased levels of MDA formation. We have concluded that antioxidant or chelating properties were not sufficient to protect sperm cells against the harmful damages of heavy metals. Flavonoids, as naturally occurring compounds may have some inhibitory effects on enzyme activities (49) or exert their growth inhibitory activities through binding to human receptors (61). Therefore, it is essential to know the exact mechanisms of metal-induced toxicity and the properties of flavonoids before prescribing medications to combat the adverse effects of heavy metals on infertility. 

## References

[1] Nordberg GF, Fowler BA, Nordberg M, Friberg L (2007). Handbook on the toxicology of metals.

[2] (1992).

[3] El-Demerdash FM, Yousef MI, Kedwany FS, Baghdadi HH (2004). Cadmium-induced changes in lipid peroxidation, blood hematology, biochemical parameters and semen quality of male rats: protective role of vitamin E and betacarotene. Food Chem Toxicol.

[4] Heim KE, Tagliaferro AR, Bobilya DJ (2002). Flavonoid antioxidants: chemistry, metabolism and structure-activity relationships. J Nutr Biochem.

[5] Plaza M, Pozzo T, Liu J, Gulshan Ara KZ, Turner C, Nordberg Karlsson E (2014). Substituent effects on in vitro antioxidizing properties, stability and solubility in flavonoids. J Agric Food Chem.

[6] Rice-Evans C (2001). Flavonoid antioxidants. Curr Med Chem.

[7] Flora SJ (2009). Structural, chemical and biological aspects of antioxidants for strategies against metal and metalloid exposure. Oxid Med Cell Longev.

[8] Mazur A, Bayle D, Lab C, Rock E, Rayssiguier Y (1999). Inhibitory effect of procyanidin-rich extracts on LDL oxidation in vitro. Atherosclerosis.

[9] Kondo K, Hirano R, Matsumoto A, Igarashi O, Itakura H (1996). Inhibition of LDL oxidation by cocoa. Lancet.

[10] Korkina LG, Afanas'ev IB (1997). Antioxidant and chelating properties of flavonoids. Adv Pharmacol.

[11] Harborne JB, Williams CA (2000). Advances in flavonoid research since 1992. Phytochemistry.

[12] Hammerstone JF, Lazarus SA, Schmitz HH (2000). Procyanidin content and variation in some commonly consumed foods. J Nutr.

[13] Carando S, Teissedre PL, Pascual-Martinez L, Cabanis JC (1999). Levels of flavan-3-ols in French wines. J Agric Food Chem.

[14] Prior RL, Cao G (1999). Antioxidant capacity and polyphenolic components of teas: implications for altering in vivo antioxidant status. Proc Soc Exp Biol Med.

[15] Kumar S, Pandey AK (2013). Chemistry and biological activities of flavonoids: an overview. ScientificWorldJournal.

[16] Mora A, Payá M, Ríos JL, Alcaraz MJ (1990). Structure-activity relationships of polymethoxyflavones and other flavonoids as inhibitors of non-enzymic lipid peroxidation. Biochem Pharmacol.

[17] Takabe W, Niki E, Uchida K, Yamada S, Satoh K, Noguchi N (2001). Oxidative stress promotes the development of transformation: involvement of a potent mutagenic lipid peroxidation product, acrolein. Carcinogenesis.

[18] Sastre J, Pallardó FV, Viña J (2000). Mitochondrial oxidative stress plays a key role in aging and apoptosis. IUBMB Life.

[19] Kawanishi S, Hiraku Y, Oikawa S (2001). Mechanism of guaninespecific DNA damage by oxidative stress and its role in carcinogenesis and aging. Mutat Res.

[20] Sheweita SA, Tilmisany AM, Al-Sawaf H (2005). Mechanisms of male infertility: role of antioxidants. Curr Drug Metab.

[21] Thomas SR, Davies MJ, Stocker R (1998). Oxidation and antioxidation of human low-density lipoprotein and plasma exposed to 3-morpholinosydnonimine and reagent peroxynitrite. Chem Res Toxicol.

[22] Moore KP, Darley-Usmar V, Morrow J, Roberts LJ 2nd (1995). Formation of F2-isoprostanes during oxidation of human low-density lipoprotein and plasma by peroxynitrite. Circ Res.

[23] Wang W, Sawicki G, Schulz R (2002). Peroxynitrite-induced myocardial injury is mediated through matrix metalloproteinase-2. Cardiovasc Res.

[24] Cooper TG, Noonan E, von Eckardstein S, Auger J, Baker HW, Behre HM (2010). World Health Organization reference values for human semen characteristics. Hum Reprod Update.

[25] Farrell PB, Foote RH, Simkin ME, Clegg ED, Wall RJ (1993). Relationship of semen quality, number of sperm inseminated, and fertility in rabbits. J Androl.

[26] World Health Organization (2001). [Laboratory manual of the WHO for the examination of human semen and spermcervical mucus interaction]. Ann Ist Super Sanita.

[27] Buege JA, Aust SD (1978). Microsomal lipid peroxidation. Methods Enzymol.

[28] Janero DR (1990). Malondialdehyde and thiobarbituric acid-reactivity as diagnostic indices of lipid peroxidation and peroxidative tissue injury. Free Radic Biol Med.

[29] Dawson EB, Ritter S, Harris WA, Evans DR, Powell LC (1998). Comparison of sperm viability with seminal plasma metal levels. Biol Trace Elem Res.

[30] Yousef MI, Salama AF (2009). Propolis protection from reproductive toxicity caused by aluminium chloride in male rats. Food Chem Toxicol.

[31] Yousef MI, El-Morsy AM, Hassan MS (2005). Aluminium-induced deterioration in reproductive performance and seminal plasma biochemistry of male rabbits: protective role of ascorbic acid. Toxicology.

[32] Waalkes MP, Anver M, Diwan BA (1999). Carcinogenic effects of cadmium in the noble (NBL/Cr) rat: induction of pituitary, testicular, and injection site tumors and intraepithelial proliferative lesions of the dorsolateral prostate. Toxicol Sci.

[33] Waalkes MP, Anver MR, Diwan BA (1999). Chronic toxic and carcinogenic effects of oral cadmium in the Noble (NBL/ Cr) rat: induction of neoplastic and proliferative lesions of the adrenal, kidney, prostate, and testes. J Toxicol Environ Health A.

[34] Oliveira H, Spanò M, Santos C, Pereira Mde L (2009). Adverse effects of cadmium exposure on mouse sperm. Reprod Toxicol.

[35] Monsefi M, Alaee S, Moradshahi A, Rohani L (2010). Cadmiuminduced infertility in male mice. Environ Toxicol.

[36] Gomes M, Gonçalves A, Rocha E, Sá R, Alves A, Silva J (2015). Effect of in vitro exposure to lead chloride on semen quality and sperm DNA fragmentation. Zygote.

[37] Yousef MI, Kamel KI, El-Guendi MI, El-Demerdash FM (2007). An in vitro study on reproductive toxicity of aluminium chloride on rabbit sperm: the protective role of some antioxidants. Toxicology.

[38] Farombi EO, Adedara IA, Akinrinde SA, Ojo OO, Eboh AS (2012). Protective effects of kolaviron and quercetin on cadmiuminduced testicular damage and endocrine pathology in rats. Andrologia.

[39] Gibb Z, Butler TJ, Morris LH, Maxwell WM, Grupen CG (2013). Quercetin improves the postthaw characteristics of cryopreserved sex-sorted and nonsorted stallion sperm. Theriogenology.

[40] Metodiewa D, Jaiswal AK, Cenas N, Dickancaite E, Segura-Aguilar J (1999). Quercetin may act as a cytotoxic prooxidant after its metabolic activation to semiquinone and quinoidal product. Free Radic Biol Med.

[41] Zribi N, Chakroun NF, Elleuch H, Abdallah FB, Ben Hamida AS, Gargouri J (2011). Sperm DNA fragmentation and oxidation are independent of malondialdheyde. Reprod Biol Endocrinol.

[42] Rafique M, Khan N, Perveen K, Naqvi A (2009). The effects of lead and zinc on the quality of semen of albino rats. J Coll Physicians Surg Pak.

[43] Graça A, Ramalho-Santos J, de Lourdes Pereira M (2004). Effect of lead chloride on spermatogenesis and sperm parameters in mice. Asian J Androl.

[44] Mushina EV (1989). Study of the combined effects of lead and cadmium on experimental animals. Gig Sanit.

[45] Sikka SC (2001). Relative impact of oxidative stress on male reproductive function. Curr Med Chem.

[46] Ige SF, Akhigbe RE (2012). The role of Allium cepa on aluminuminduced reproductive dysfunction in experimental male rat models. J Hum Reprod Sci.

[47] Aitken RJ (1995). Free radicals, lipid peroxidation and sperm function. Reprod Fertil Dev.

[48] Moretti E, Mazzi L, Terzuoli G, Bonechi C, Iacoponi F, Martini S (2012). Effect of quercetin, rutin, naringenin and epicatechin on lipid peroxidation induced in human sperm. Reprod Toxicol.

[49] Khanduja KL, Verma A, Bhardwaj A (2001). Impairment of human sperm motility and viability by quercetin is independent of lipid peroxidation. Andrologia.

[50] Breitbart H, Rubinstein S, Nass-Arden L (1985). The role of calcium and Ca2+-ATPase in maintaining motility in ram spermatozoa. J Biol Chem.

[51] Iavicoli I, Fontana L, Bergamaschi A (2009). The effects of metals as endocrine disruptors. J Toxicol Environ Health B Crit Rev.

[52] Pizent A, Tariba B, Živković T (2012). Reproductive toxicity of metals in men. Arh Hig Rada Toksikol.

[53] Ghaffari MA, Motlagh B (2011). In vitro effect of lead, silver, tin, mercury, indium and bismuth on human sperm creatine kinase activity: a presumable mechanism for men infertility. Iran Biomed J.

[54] Järup L (2003). Hazards of heavy metal contamination. Br Med Bull.

[55] Yoshida M, Kawano N, Yoshida K (2008). Control of sperm motility and fertility: diverse factors and common mechanisms. Cell Mol Life Sci.

[56] McLaren JF (2012). Infertility evaluation. Obstet Gynecol Clin North Am.

[57] Lavranos G, Balla M, Tzortzopoulou A, Syriou V, Angelopoulou R (2012). Investigating ROS sources in male infertility: a common end for numerous pathways. Reprod Toxicol.

[58] Niederberger C (2012). Re: the role of sperm oxidative stress in male infertility and the significance of oral antioxidant therapy. J Urol.

[59] Mansuri ML, Parihar P, Solanki I, Parihar MS (2014). Flavonoids in modulation of cell survival signalling pathways. Genes Nutr.

[60] Hu JP, Calomme M, Lasure A, De Bruyne T, Pieters L, Vlietinck A (1995). Structure-activity relationship of flavonoids with superoxide scavenging activity. Biol Trace Elem Res.

[61] Garrett SD, Lee HA, Morgan MR (1999). A nonisotopic estrogen receptor-based assay to detect estrogenic compounds. Nat Biotechnol.

